# Total and Sustainable Utilization of Biomass Resources: A Perspective

**DOI:** 10.3389/fbioe.2020.00546

**Published:** 2020-06-05

**Authors:** Quang A. Nguyen, William A. Smith, Bradley D. Wahlen, Lynn M. Wendt

**Affiliations:** Idaho National Laboratory, Idaho Falls, ID, United States

**Keywords:** biomass, feedstock, preprocessing, co-products, conversion-ready feedstock, biorefinery, depot, corn stover fractionation

## Abstract

Feedstock cost is a major variable cost component in conversion to biofuels and chemicals. Consistent feedstock quality is critically important to achieve high product yield and maximum onstream time. Traditionally, raw biomass materials are delivered directly to the biorefineries where they are preprocessed to feedstock prior to being converted to products. Since many types of biomass materials—including agricultural residues, energy crops, and logging residues—are harvested according to growth cycles and optimal harvesting time, just-in-time steady supply of raw biomass to the biorefineries is not possible. Instead, biomass materials are stored, then delivered to the biorefineries as needed. Experience to date indicates that this approach has caused many issues related to logistics, biomass losses due to microbial degradation and fire, and inconsistent feedstock quality due to variability in the properties of as-delivered biomass. These factors have led to high feedstock cost, low throughput, and low product yield for the biorefineries. Idaho National Laboratory has developed a new strategy to address the problems encountered in the traditional approach in biomass feedstock supply, storage, and preprocessing mentioned above. The key components of this strategy are (1) preservation and preconditioning of biomass during storage, (2) utilization of all the biomass, including minor components that are normally considered wastes or contaminants, and (3) maximization of the value of each component. This new approach can be accomplished using feedstock preprocessing depots located near the biomass-production sources.

## Introduction

This paper focuses primarily on utilization of agricultural residues, specifically corn stover, and herbaceous energy crops. Feedstock cost is the largest manufacturing-cost component in cellulosic biofuel production. The National Renewable Energy Laboratory (NREL) has projected that, to meet the DOE fuel selling price target of $2.50/GGE by 2030, the price of biomass feedstock delivered to the reactor throat for a biomass-to-hydrocarbon fuels biochemical conversion facility must be < $71.3/dry short ton (2016 U.S. dollars) (Davis et al., 2018). Based on experience at pioneer biorefineries, this target price would be very difficult to meet using current technology and only applicable in certain locations where low-cost biomass is available. In addition to obtaining low-cost feedstock, another major issue with the conventional approach is that preprocessing of raw biomass materials (especially baled agricultural residues) is difficult and often leads to low equipment uptime. The major challenges identified by industry include biomass-feedstock flowability, variability in feedstock properties, lack of equipment-performance data, and lack of standard feedstock specifications (US DOE, 2016). Additionally, integrating feedstock preprocessing with biofuel conversion in a single facility lowers plant productivity as operational issues in the preprocessing area often cause shutdown of downstream conversion-unit operations.

One approach in reducing the cost of delivered biomass feedstock is to blend high-carbohydrate biomass (e.g., two-pass corn stover or switchgrass) and low-cost biomass (e.g., grass clippings) to achieve a projected cost of $79.1/dry short ton (2016 U.S. dollars) by 2022 (Roni et al., 2018). The blended biomass materials are pelleted to facilitate high-density storage and shipping as well as improved handling characteristics at the biorefinery. However, the blending approach is restricted to areas where the low-cost and high-carbohydrate-content biomass materials are available. Another issue with supplying biomass feedstock with highly variable component (carbohydrates and lignin) concentrations is that a biorefinery capable of converting these major components to biofuels and high-value coproducts would be complex and require high capital investment, which is a significant barrier to commercialization, especially for new technologies. The capital cost of a 50 million annual gallons cellulosic ethanol plant is estimated at $4.30 (1999 dollars) per annual gallon, compared to about $1.25 per annual gallon for a dry-grind corn-ethanol plant (McAloon et al., 2000).

Idaho National Laboratory is investigating a new approach through which advanced feedstock depots preserve biomass and convert raw biomass into several conversion-ready feedstocks, targeting a wide range of markets, including biofuels, bioproducts, animal feed, and agriculture. In this way, the potential value of preprocessed biomass material is higher than for single-use and, with a larger customer base, the financial risk of such feedstock depots would be reduced. The conversion-ready feedstocks can also be tailored to end users' specifications. This approach combines in-storage preconditioning of biomass to minimize microbial degradation of carbohydrates with fractionation to produce high-value products. The following sections provide insights into key components of an advanced feedstock preprocessing depot that would produce many benefits: simplified biomass supply logistics, in-storage preconditioning, and product fractionation and recovery.

## Biomass Supply and Logistics

Corn stover has been identified as the most abundantly available agricultural residue suitable for conversion to biofuels and chemicals (Langholtz et al., 2016). In the agriculture sector, the two most common methods of storing agricultural residue are (1) square or round bales and (2) ensiled piles or bunkers. Assuming an average dry mass per large square bale is 500 kg, a 2,000-metric ton/day facility will consume 4,000 bales per day, not counting dry matter losses during storage and preprocessing. The normal inventory of a biorefinery is 5 days. For a 2,000 metric tons/day facility, the storage area for a 5-day inventory is at least 5.9 hectares (14.5 acres), assuming each 2,000-bale stack is 7-bale high and spaced 60 m apart to minimize the risk of fire spreading from one stack to another (Webb et al., 2018). Because the harvesting period for corn stover averages 4 months, storing an 8-month supply of bales in satellite storage requires an area of at least 256 hectares (633 acres). Handling and transporting bales from field to satellite storage then to biorefineries significantly add to the cost of feedstock. Storage of biomass bales (e.g., corn stover) has many problems that lead to high dry matter loss, variability in feedstock properties, and high cost. A 2,000-bale stack (7 bales high) requires up to 59% of the bales to have at least one side exposed either to the ground or external air if the stack is not covered. If the top of the stack is covered, up to 45% of the bales are exposed. Moisture movement in exposed bales leads to higher degradation and variable properties (Smith et al., 2013). Moisture and ash were identified as the major properties that have significant impact on the operability of feedstock-preprocessing equipment (US DOE, 2016). High moisture and multiple layers (flakes) of corn stover bales reduce the throughput of bale grinders, cause surge flows, and result in variable particle size (Nguyen, 2019). Multipass baling logistics lead to high extrinsic ash content due to soil contamination (Bonner et al., 2014). Another issue with bale logistics is the large amount of polypropylene twine (for square bales) and polyethylene bale net wrap (for round bales) requiring disposal. It is estimated that a 2,000-metric ton/day biorefinery using square bales generates about 8.4 million pieces of 6.7 m-long twine every year of operation. Use or recycling this waste could be problematic for many rural locations. Most large square balers leave several pieces of twine (tailings), each 2–4 cm long, on the bales. These contaminants are difficult to detect and remove. Removal of bale twine and net wrap using current mechanical technology is <100% successful. Twine and net-wrap contaminants can cause clogging of piping and equipment (Sluska and Bushong, 2019).

The heterogenous components of corn stover (stalk, leaf, husk, cob) and softwood logging residuals (white wood, bark, twigs, needles) lead to variable physical and mechanical properties and chemical compositions. These variabilities result in material-handling and operational problems, lower throughput and product yields in pioneer biorefineries (US DOE, 2016). Fractionating biomass into major components and converting them to conversion-ready feedstock is one way to address the material-handling and conversion-yield issues.

**Figure 1:**
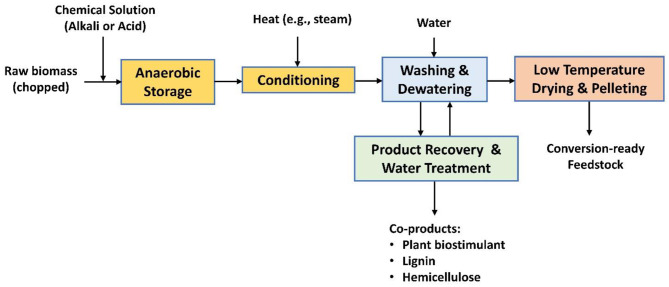
High-moisture anaerobic storage and fractionation of biomass.

High-moisture (40–65% wet weight basis), anaerobic storage of feedstock has many advantages over bale storage, including lower dry matter loss (Wendt et al., 2018), much lower fire risk, and lower cost of handling. It also provides the opportunity to carry out leaching or microbial or chemical preconditioning during storage. Compared to bale storage, pile storage requires a significantly smaller storage area: a 5-day inventory pile for a 2,000 metric ton/day facility only needs about 0.64 hectare (1.58 acre) assuming a recommended average compacted bulk density of 240 kg/m^3^ (15 lb/ft^3^) dry basis to achieve low dry matter loss (Holmes and Muck, 2007). The pile can be located next to the biorefinery, and the feedstock can be conveyed from the storage area into the plant. This results in lower handling costs. Biomass-chopping logistics (Mann et al., 2019), as opposed to baling logistics, is more suitable for high-moisture, anaerobic storage, especially for herbaceous energy crops. Chopped corn stover can be compacted using a 0.3-m-diameter auger to a relaxed bulk density of about 208 kg/m^3^ (13 lb/ft^3^) dry basis or higher (Franz, 2007). As a comparison, the bulk density of corn stover square bales is about 177 kg/m^3^ (11 lb/ft^3^), and about 141 kg/m^3^ (8.8 lb/ft^3^) dry basis for round bales. Mobile screw compactors can be used to compact chopped biomass into transporters in the field (Gruithuis et al., 2007; Jiskra et al., 2017). The compacted biomass can then be transferred to depots for preprocessing to conversion-ready densified feedstock. A possible configuration of chopped biomass logistic is shown in Figure 1. A one-pass harvester blows chopped corn stover into mobile forage wagons in the field. The wagons are pulled to the side of the field, where the chopped biomass is compacted in transporters or driven directly to nearby preprocessing depots. The forage wagons can precompact chopped biomass to 112–139 kg/m^3^ dry basis (Suokannas and Nysand, 2009). The net cost of transporting and handling of chopped corn stover has potential to be lower than that for baled corn stover because of the shorter total travel distance to the local depot and the elimination of intermediate bale-storage and handling steps. Table 1 lists the main advantages of the chopped biomass logistics over the baling logistics.

**Table 1:** Qualitative comparison between baling and chopping logistics for herbaceous biomass.

**Attributes**	**Baling Logistics**	**Chopping Logistics**
Agricultural residues harvest and collection logistics	Common	Uncommon for most uses but common for producing silage
Dry matter loss during storage outdoors for weeks & months	10–20% depending on the moisture content of the bales and the weather conditions	5–6% under anaerobic storage condition
Biomass properties	Significant variability in properties: moisture, ash, fiber integrity, particle size, chemical composition	More consistent properties than baled biomass
Fire risk	High fire risks from lighting, self-combustion and arson	Very low fire risk because of >45% moisture and anaerobic conditions
Cost of storage and handling	High because the bale stacks take a lot of space and must be stored far apart to prevent fire from spreading. The storage area for bales is approximately 9 times that for piles, plus there are many satellite bale storage sites.	Storage piles can be located next to the feedstock depots so the biomass can be conveyed into the preprocessing area. The modular depots are strategically located near the biomass sources to lower the cost transportation.
Facilitating production of multiple products including conversion-ready feedstocks?	No, as it would be very expensive.	Yes, biological and chemical treatment can be readily incorporated into the high- moisture biomass storage operation.
Impact on feedstock depot and biorefinery operations	Low operational reliability and product yield because of the high variability in biomass properties. Higher capital and operating costs for biorefineries.	Improved operational reliability and lower capital and operating costs.
Impacts by weather	Wet and cold weather may prevent field drying and baling operations. As a result, the sources of biomass supply are generally limited to dry weather areas.	Weather has less impact on harvesting and collection compared to baling operation.
Waste streams	Improper disposal of bale twines and net wrap can cause negative impacts on the environment and wide life.	No waste bale twines and net wrap. Lower environmental impact and carbon footprint.

Logging residues have also been identified in the billion-ton report as promising low-cost woody biomass. Softwood logging residues comprise mainly branches and treetops. With proper storage and preprocessing, the logging residues can be turned into a suitable feedstock for thermochemical conversion to biofuels. To minimize contamination by soil and facilitate in-field preprocessing, the logging residues should be stored in piles, and not scattered on the ground. The residues should be seasoned for about 1 year or longer to partly lower the moisture content to less than about 25% (wet basis) and facilitate defoliation (Nilsson, 2016). Pile drying to below 25% moisture content may not be practical in regions with high yearly precipitation. Generally, in the Southeastern U.S., it takes 1 year for the moisture content of logging residues to lower to about 30%, and this time requirement is longer for the Pacific Northwest U.S. Drying logging residues, however, may result in lower bark removal efficiency because the wood-to-bark adhesion strength is significantly higher at moisture content below about 40% (Chow and Obermajer, 2004). Logging residues can be sorted, screened and chipped in the field, compacted in transporters, then transferred to preprocessing depots.

## Biomass Preprocessing and Fractionation

The goal of biomass preprocessing is to produce consistent feedstock that meets conversion specifications. The heterogeneous makeup and properties of corn stover and softwood logging residues have proven to be difficult barriers to overcome using traditional preprocessing techniques, including milling, air classifying, and screening. The shear and impact forces required to break the various anatomical fractions of corn stover (rind, pith, leaf, husk, and cob) differ by tissue type (Anazodo, 1980; Zhang et al., 2016, 2017; Workiye and Woldsenbet, 2019). Applying sufficiently high impact and shear force to fracture tough components such as cob, husk and rind will pulverize the more fragile components such as pith and leaf, which leads to wide particle-size distribution with a high proportion of fines. Wide particle-size distribution has the potential to cause uneven mass flow, heat, and mass transfer in continuous high-solid pretreatment reactors. Furthermore, corn stover rind is more recalcitrant than the pith and leaf fractions (Crofcheck and Montross, 2004; Duguid et al., 2009; Zeng et al., 2012; Li et al., 2014); therefore, mild pretreatment (such as hot water and dilute sodium hydroxide pretreatment) of corn stover likely results in a compromised enzymatic-hydrolysis sugar yield.

One method to achieve high (>90% theoretical) total sugar yield after pretreatment and enzymatic hydrolysis of corn stover is to apply high-temperature, short-residence-time dilute sulfuric acid steam explosion pretreatment (Tucker et al., 2003). This pretreatment method is effective in deconstructing the recalcitrant rind fibers but does not significantly degrade more labile components such as pith, leaf and husk. This approach is analogous to the increased milling forces discussed above; increased processing intensity is used to overcome biomass heterogeneities. However, dilute acid pretreatment releases organic acids, lignin and carbohydrate degradation products that are inhibitory to enzyme and fermenting organisms (Casey et al., 2010; Jonsson et al., 2013; Qin et al., 2016).

An alternate method is to fractionate corn stover into the major anatomical components, then process them separately. This method could be economically viable if high-value uses of one or more components could be developed. As an example, bleached soda pulp can be produced from corn-stover stalks, but the presence of pith causes low yield and poor drainage (Byrd and Hurter, 2014). Depithed corn stalks are expected to improve drainage and make corn-stover pulp production more feasible. In this method, fractionation of corn stover could be performed during harvesting, before all the components are mixed and compacted together (such as in a bale); entanglement of these anatomical components makes it much more difficult to separate them. During harvest, it is possible to separate corn plants into a fraction comprising stalks and leaves and a fraction comprising husk and cobs (Shinners et al., 2009). Using screening and air classification, the leaves can be readily separated from the stalks, and the husk separated from the cobs. The separation could be performed in the field before compacting the various fractions into transporters. The stalks can potentially be de-pithed at the preprocessing depots using similar technology for de-pithing sugarcane bagasse or industrial hemp (Ren et al., 2016; Chen and Qu, 2017).

Alternately, or in addition to anatomical fractionation of biomass, chemical fractionation is possible. One fractionation option produces conversion-ready feedstock which, depending on the starting raw biomass, can be used in biochemical or thermochemical conversion processes (Figure 1). Alkali treatment, combined with mechanical deconstruction of fibers, can significantly improve the enzymatic cellulose digestibility of lignocellulosic biomass (Chen et al., 2014; Yuan et al., 2017). More than 40% of the lignin content can be extracted from corn stover to produce a conversion-ready, carbohydrate-rich fiber fraction and a lignin-rich liquor. Lignin extraction can be accomplished via alkali pre-impregnation during storage (Wendt, 2019) followed by conditioning and washing. Conditioning may include high temperatures (>50°C) and/or additional chemical treatment (e.g., with peroxide) to improve delignification (Saha and Cotta, 2014; Mittal et al., 2017). The partially delignified fiber is then washed to recover solubilized products, mechanically dewatered, deconstructed, air-dried, and then pelletized to produce a conversion-ready feedstock. The liquid fraction, a coproduct, contains solubilized carbohydrates, lignin, organic acids, minerals, and other extractives.

## Product Applications

Pioneer biorefineries based on enzymatic hydrolysis technology generally utilized lignin remaining after fermentation as fuel in a biomass boiler. This lignin-utilization method is of low value because of the low heat content of high-moisture (about 50% wet weight basis) lignin cake. The extractives, organic acids, phenolics, and inorganics require remediation or waste treatment before disposal, which results in increased operational complexity and costs.

Feedstock preprocessing depots utilizing fractionation processes can produce multiple diverse products serving a wide customer base including biofuel producers, biochemical, biomass powers, agriculture, horticulture, and animal feed. Conversion-ready feedstocks in flowable pellet form will improve the operational reliability, reduce capital, and operating costs of biorefineries. Feedstock depots generate no waste stream of bale polypropylene twine or polyethylene net wrap when using chopped biomass logistics.

The liquid stream can be further fractionated to recover a lignin powder product (via acid precipitation and filtration) and a liquid product containing other soluble components (carbohydrates, organic acids, phenolics, extractives, and inorganics). The liquid product could potentially be used as a biostimulant to promote plant growth.

Alkali lignin can be used as substitute for phenol in lignin-based phenolic-resin applications for manufacturing of composite wood products (Ghorbani et al., 2016; Zafar et al., 2019), lignin-based polymers (Naskar and Tran, 2017; Ganewatta et al., 2019), or as antioxidant and antimicrobial agents (Spiridon, 2018) or converted to fuels and chemicals (Beckham, 2019; Ha et al., 2019).

Resin and fatty acids can be extracted from low-value fractions of logging residues such as needles, bark, and small branches (Eriksson et al., 2018).

## Conclusion

The current agricultural practice of multipass harvesting, collection, and baling of herbaceous biomass is not suitable for providing feedstock that meets the required specification of biorefineries without expensive and complex preprocessing methods to produce consistent quality feedstock from raw biomass with high variability of properties. Single-pass chopped biomass logistics with in-field compaction, combined with chemically treated anaerobic storage, will not only minimize soil contamination and eliminate bale twine and net wrap contaminants, but also lead to viable options for fractionating biomass to useful products and minimize waste streams. Furthermore, a preprocessing depot can be flexibly configured to produce conversion-ready feedstocks that meet specific qualities for individual conversion technologies. It is envisioned that feedstock depots can supply conversion-ready feedstocks to a variety of conversion technologies and create a wide range of coproducts so that these depots would operate as profitable businesses, not dependent on a single biorefinery. This approach has the potential to lower technical and economic barriers to growing a biobased economy. Early adopters of multiproduct feedstock depots include biomass-feedstock integrators and suppliers, feed aggregators, biomass-pellet producers, and wood-mulch producers. They are already in the business of biomass preprocessing and serve well-established industries, such as wood products, pulp and paper, biomass power plants, feed lots, and horticultural sectors. It would be an incentive for these biomass-feedstock producers to expand their product portfolio into higher-value products such as conversion-ready feedstocks, lignin, and extractives.

## Author Contributions

QN, WS, BW, and LW prepared this manuscript. QN and LW contributed conception and design of the study. QN wrote the first draft of the manuscript. WS contributed to the Introduction and Biomass Supply and Logistics section. BW and LW contributed to the Biomass Preprocessing and Fractionation section. All authors contributed to the Product Applications section and manuscript revision, reading and approving the submitted version.

## Conflict of Interest

The authors declare that the research was conducted in the absence of any commercial or financial relationships that could be construed as a potential conflict of interest.
